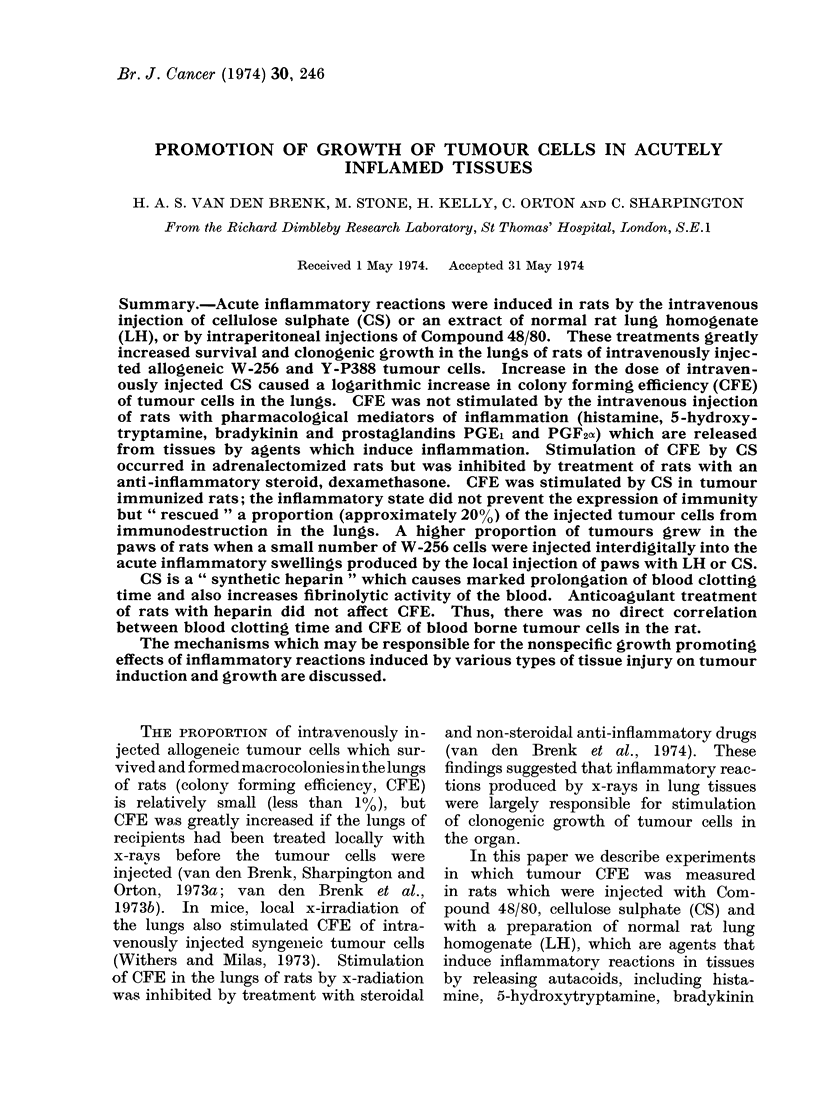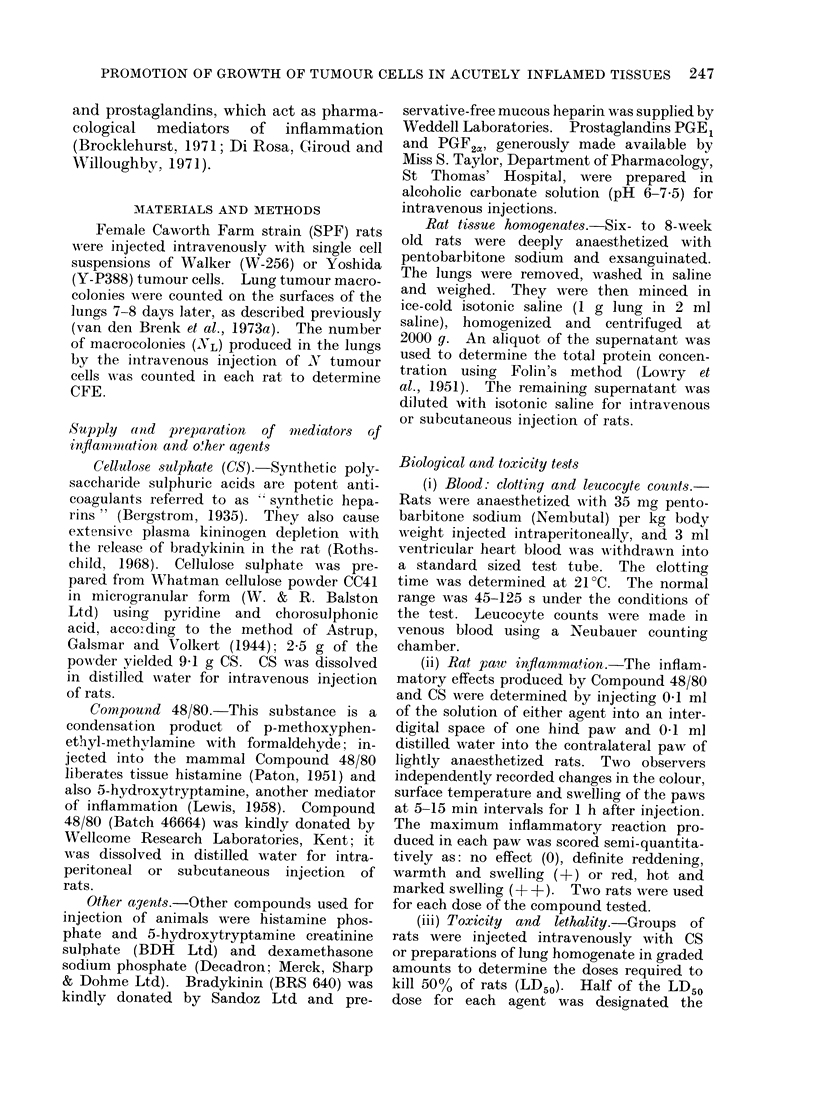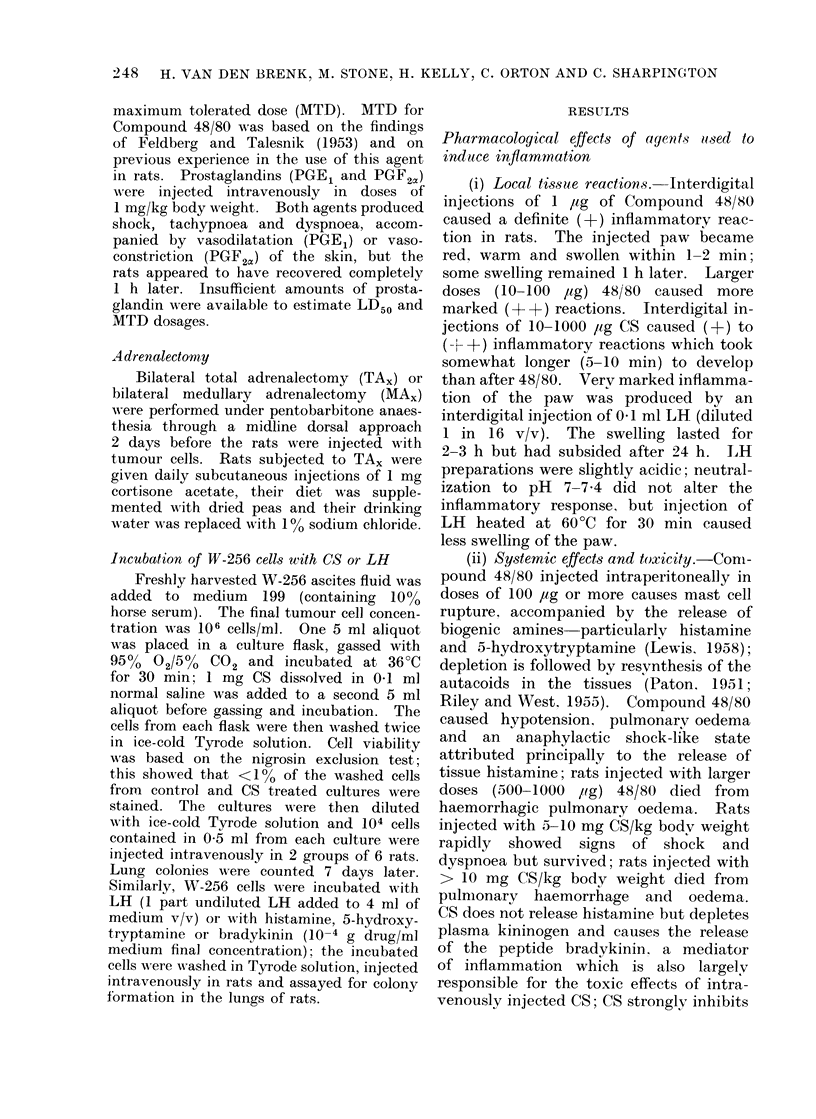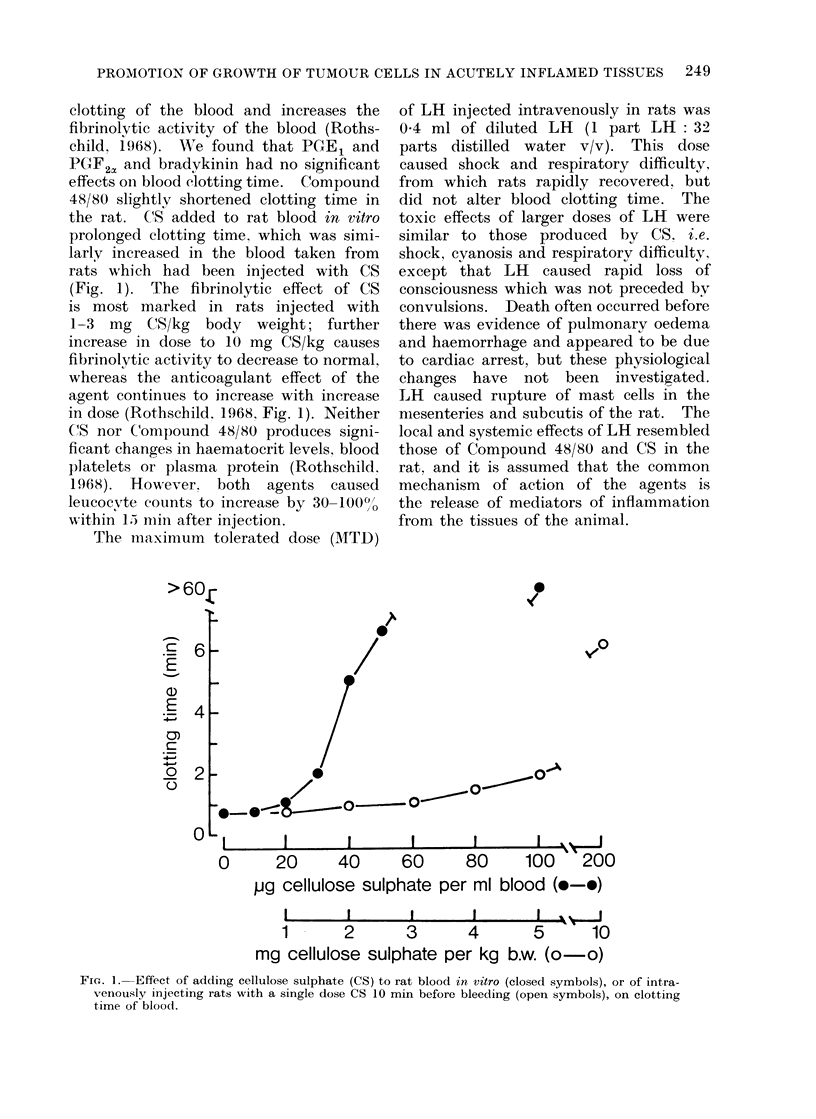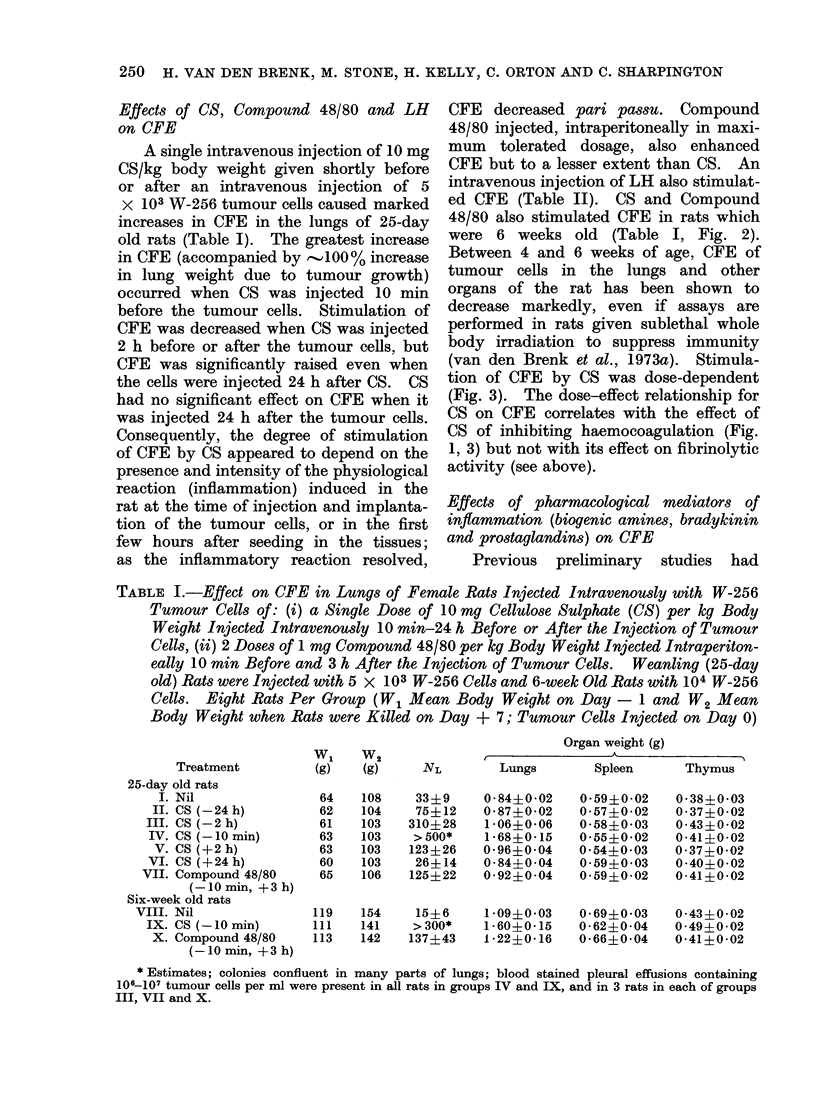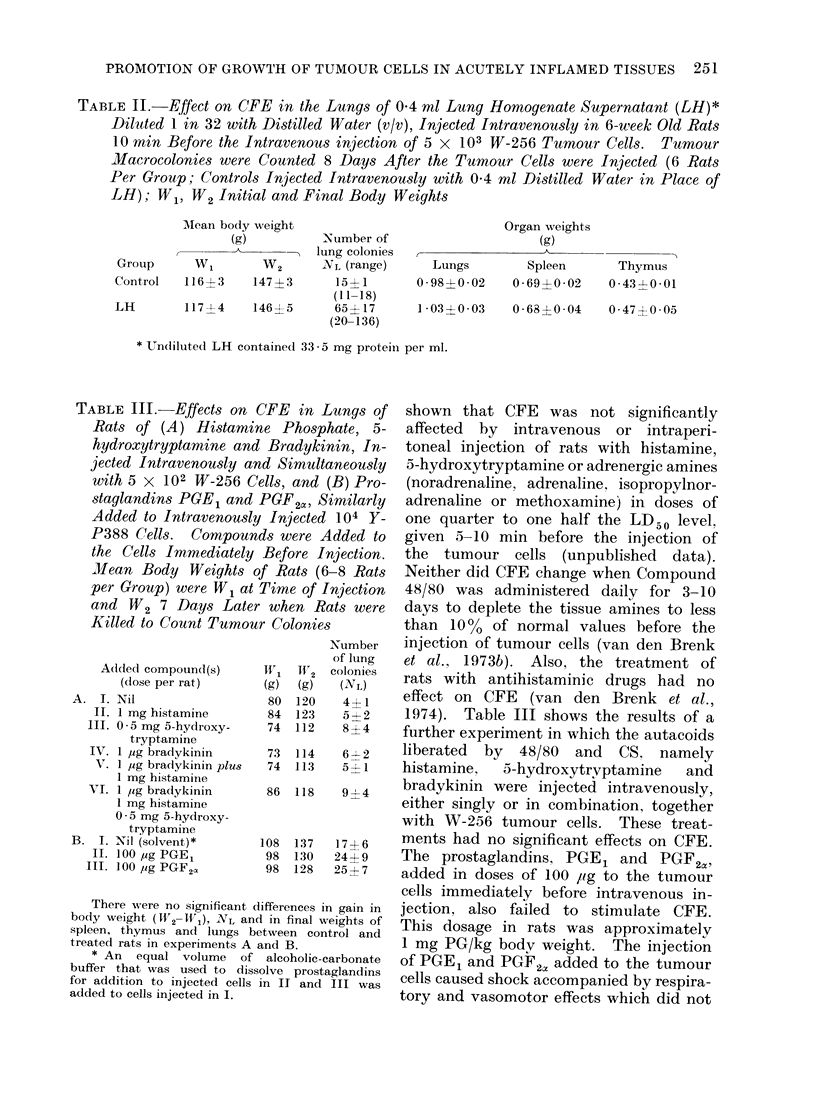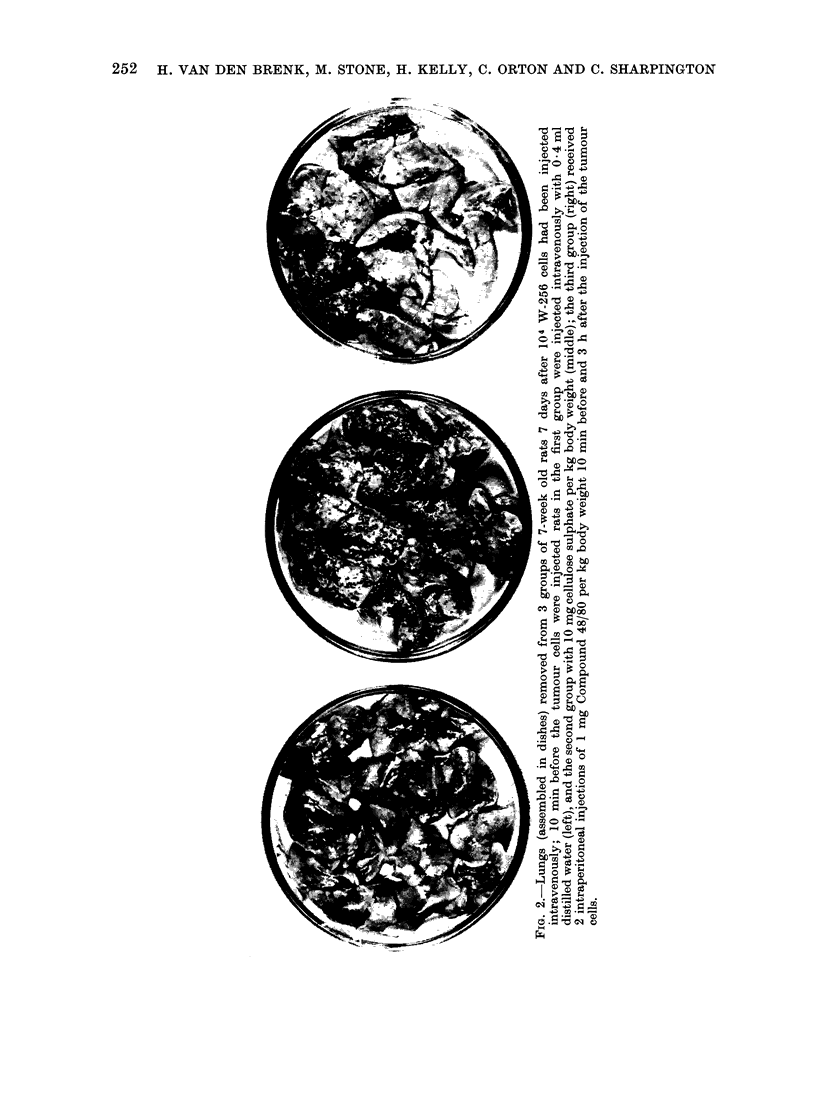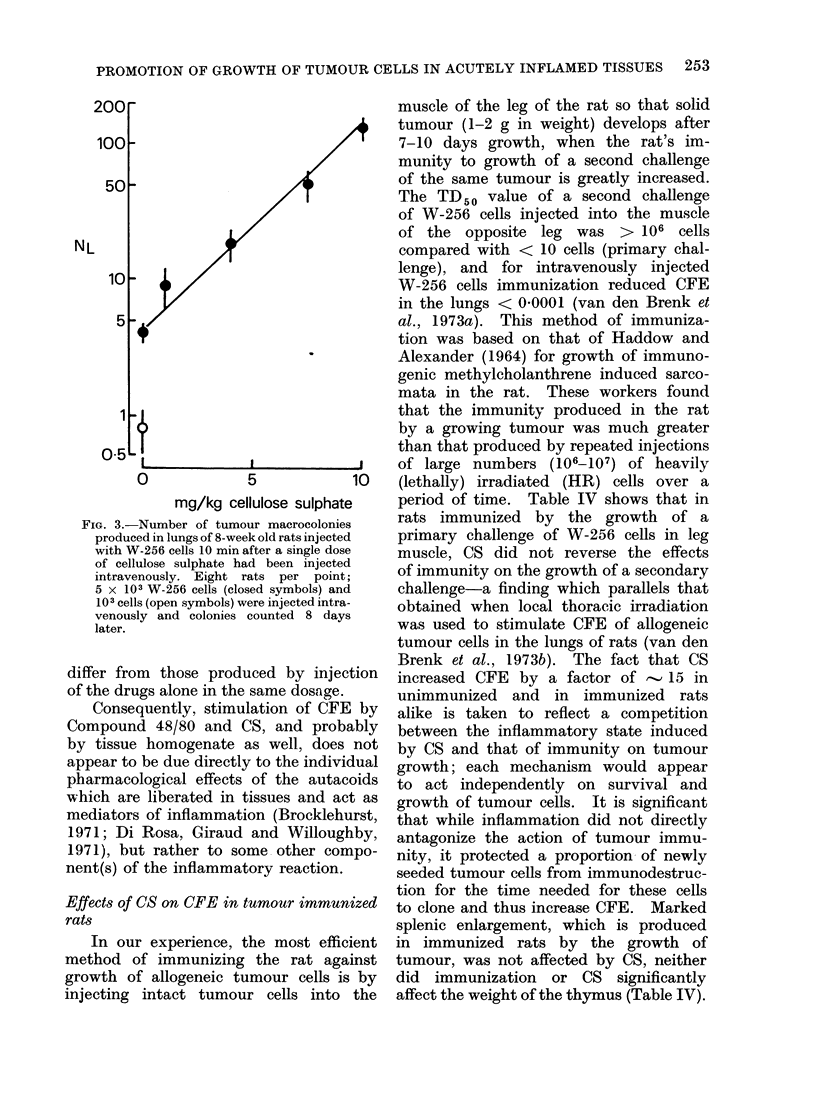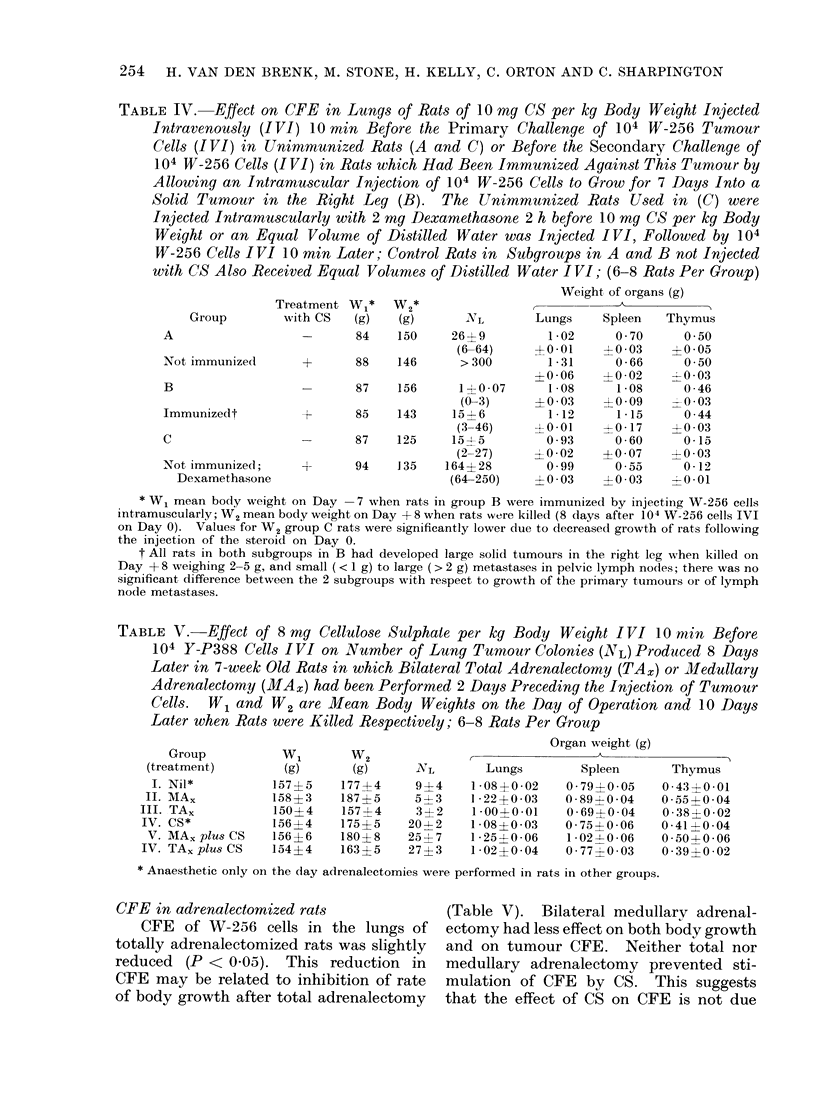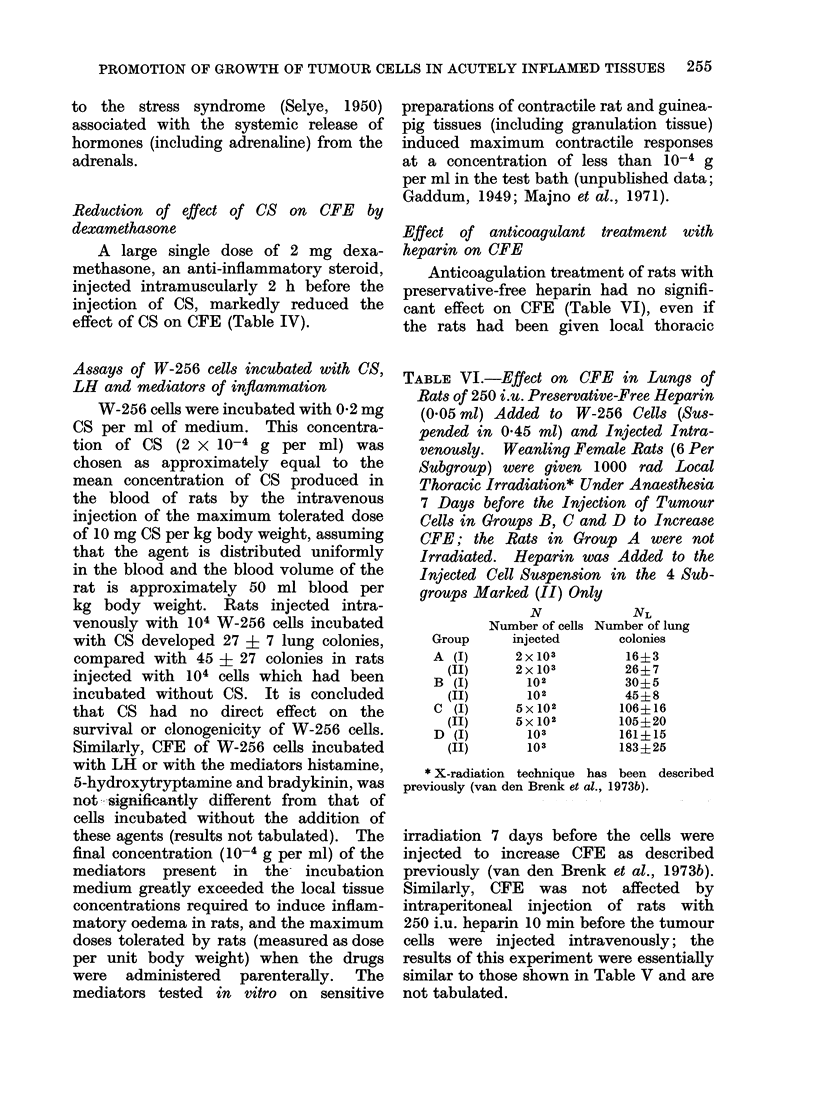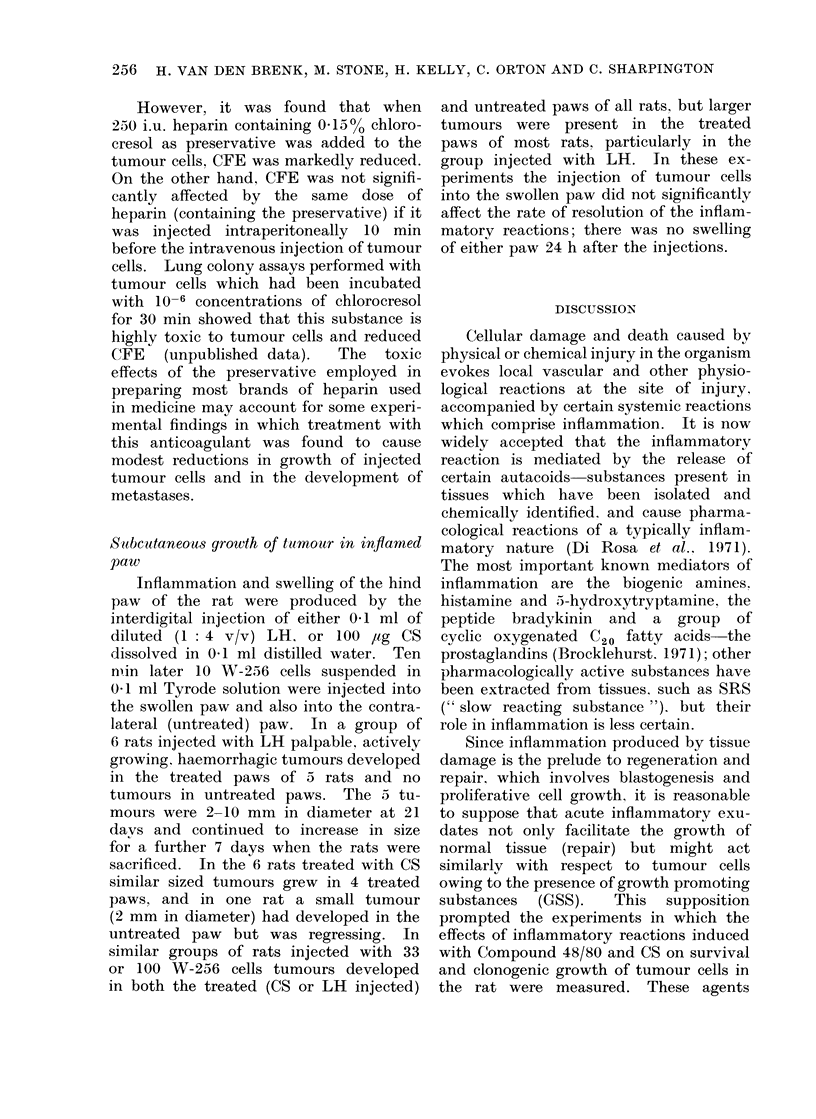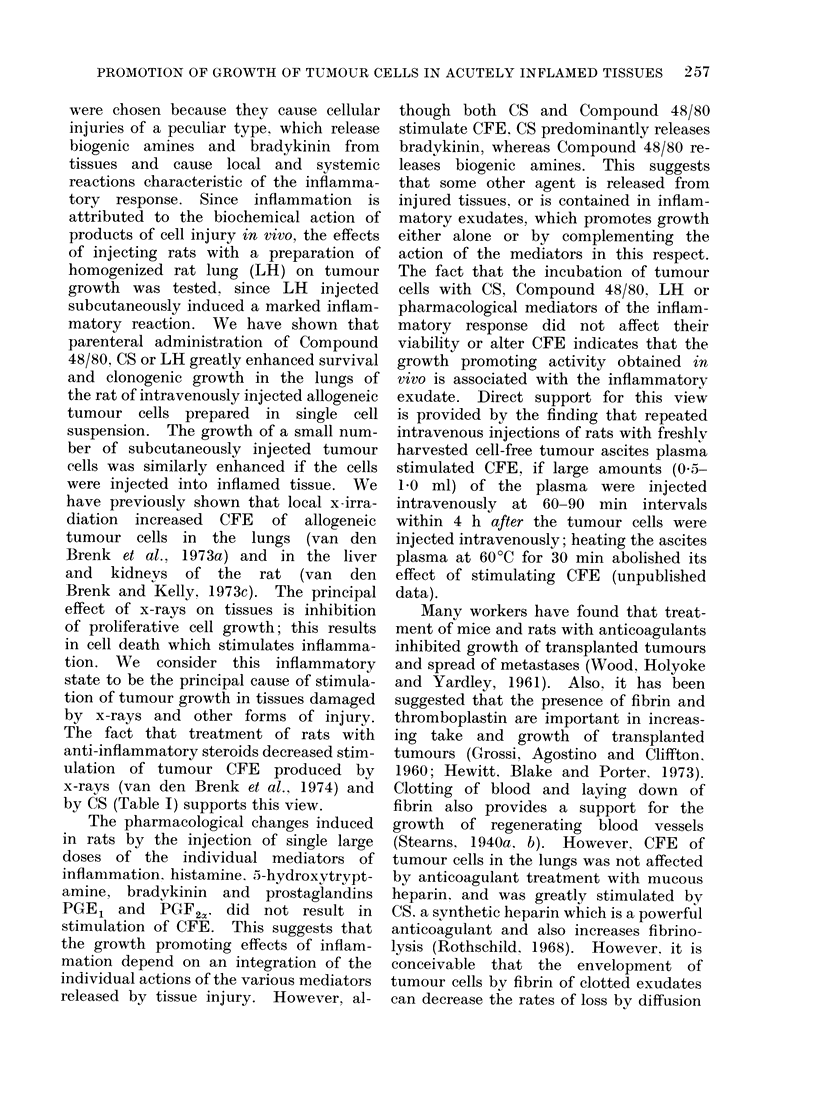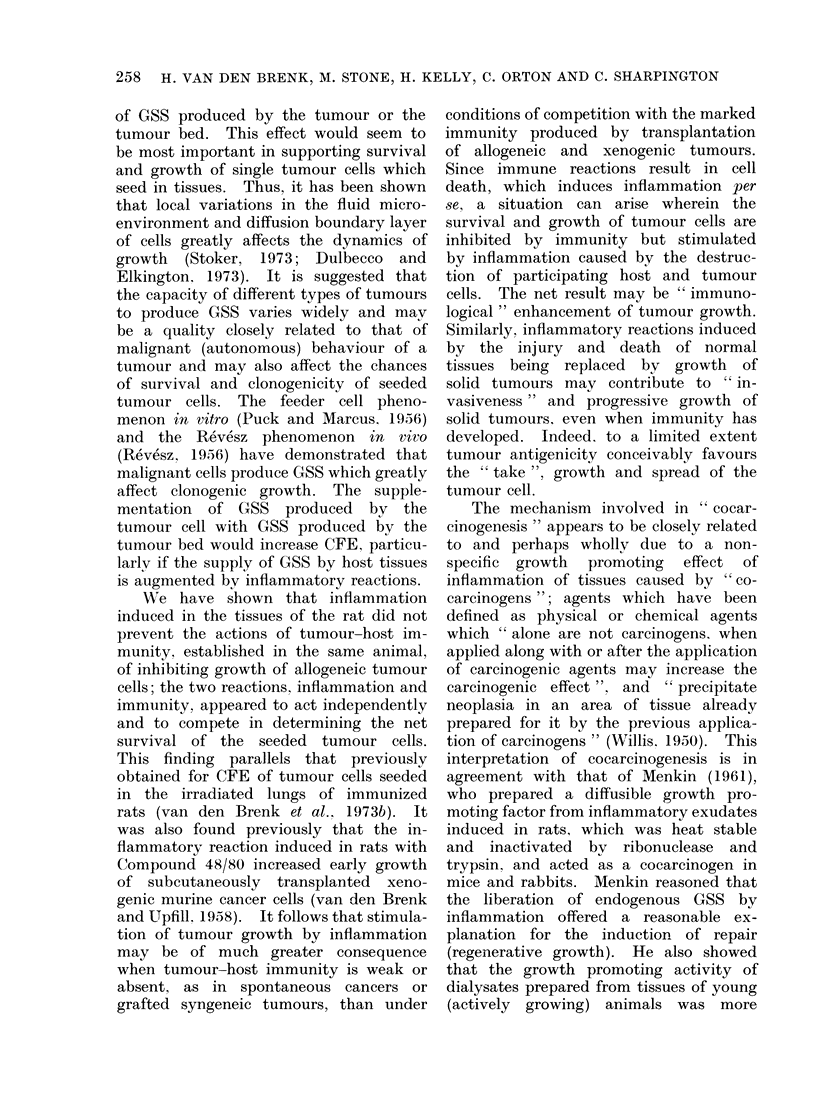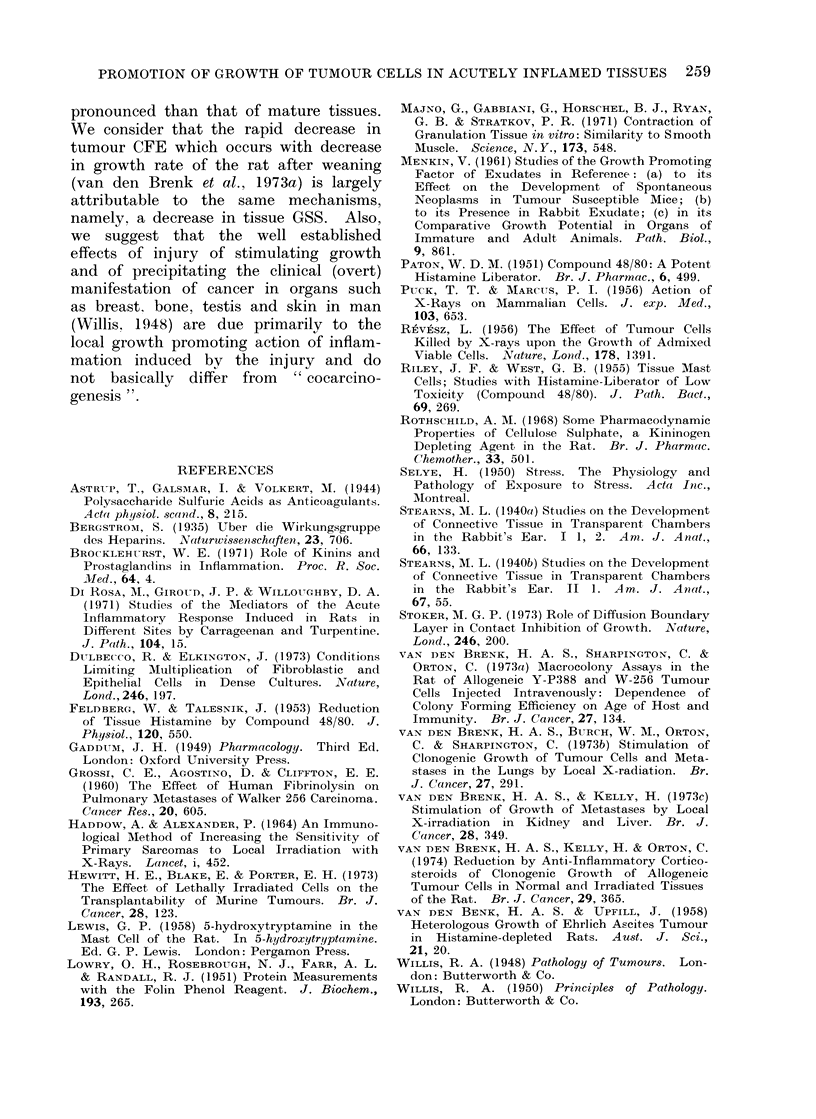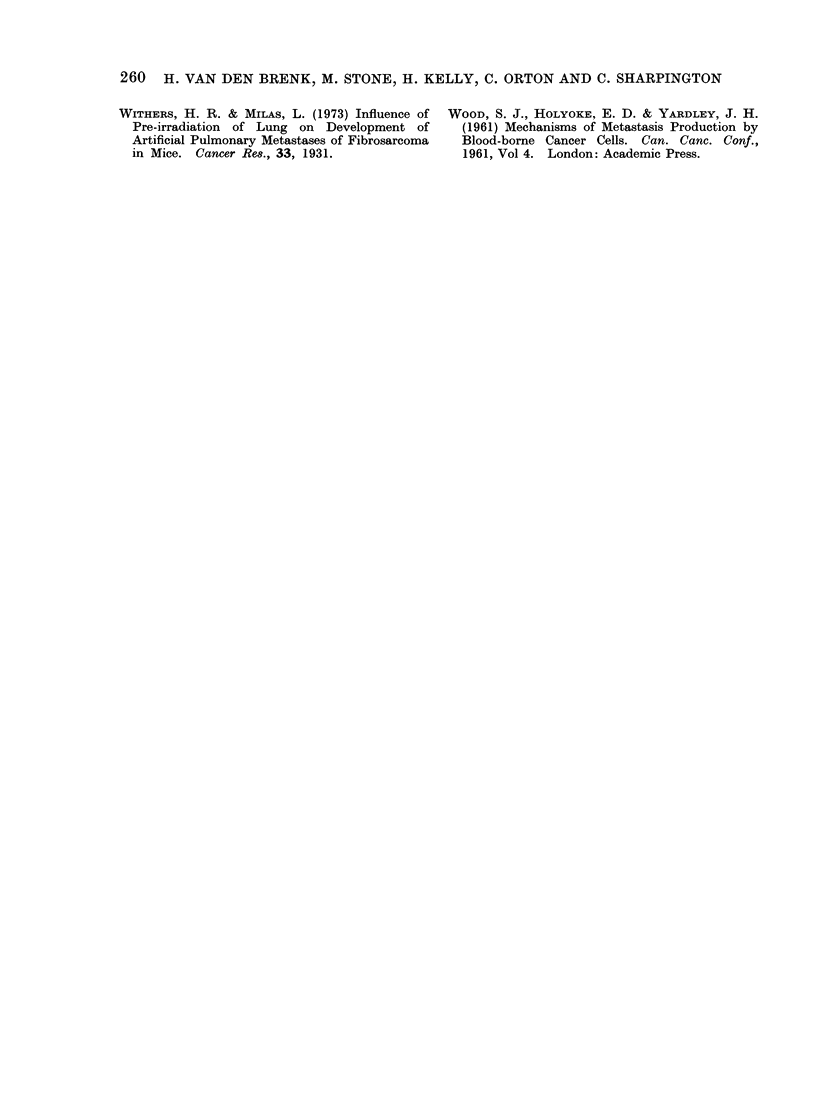# Promotion of Growth of Tumour Cells in Acutely Inflamed Tissues

**DOI:** 10.1038/bjc.1974.189

**Published:** 1974-09

**Authors:** H. A. S. van den Brenk, M. Stone, H. Kelly, C. Orton, C. Sharpington

## Abstract

**Images:**


Br. J. Cancer (1974) 30, 246

### PROMOTION OF GROWTH OF TUMOUR CELLS IN ACUTELY INFLAMED TISSUES

H. A. S. VAN DEN BRENK, M. STONE, H. KELLY, C. ORTON AND C. SHARPINGTON

*From the Richard Dimbleby Research Laboratory, St Thomas' Hospital, London, S.E.1*

Received 1 May 1974. Accepted 31 May 1974

**Summary.—Acute inflammatory reactions were induced in rats by the intravenous injection of cellulose sulphate (CS) or an extract of normal rat lung homogenate (LH), or by intraperitoneal injections of Compound 48/80. These treatments greatly increased survival and clonogenic growth in the lungs of rats of intravenously injected allogeneic W-256 and Y-P388 tumour cells. Increase in the dose of intravenously injected CS caused a logarithmic increase in colony forming efficiency (CFE) of tumour cells in the lungs. CFE was not stimulated by the intravenous injection of rats with pharmacological mediators of inflammation (histamine, 5-hydroxytryptamine, bradykinin and prostaglandins $PGE_1$ and $PGF_{2\alpha}$) which are released from tissues by agents which induce inflammation. Stimulation of CFE by CS occurred in adrenalectomized rats but was inhibited by treatment of rats with an anti-inflammatory steroid, dexamethasone. CFE was stimulated by CS in tumour immunized rats; the inflammatory state did not prevent the expression of immunity but " rescued " a proportion (approximately 20%) of the injected tumour cells from immunodestruction in the lungs. A higher proportion of tumours grew in the paws of rats when a small number of W-256 cells were injected interdigitally into the acute inflammatory swellings produced by the local injection of paws with LH or CS.**

**CS is a " synthetic heparin " which causes marked prolongation of blood clotting time and also increases fibrinolytic activity of the blood. Anticoagulant treatment of rats with heparin did not affect CFE. Thus, there was no direct correlation between blood clotting time and CFE of blood borne tumour cells in the rat.**

**The mechanisms which may be responsible for the nonspecific growth promoting effects of inflammatory reactions induced by various types of tissue injury on tumour induction and growth are discussed.**

THE PROPORTION of intravenously injected allogeneic tumour cells which survived and formed macrocolonies in the lungs of rats (colony forming efficiency, CFE) is relatively small (less than 1%), but CFE was greatly increased if the lungs of recipients had been treated locally with x-rays before the tumour cells were injected (van den Brenk, Sharpington and Orton, 1973*a*; van den Brenk *et al.*, 1973*b*). In mice, local x-irradiation of the lungs also stimulated CFE of intravenously injected syngeneic tumour cells (Withers and Milas, 1973). Stimulation of CFE in the lungs of rats by x-radiation was inhibited by treatment with steroidal and non-steroidal anti-inflammatory drugs (van den Brenk *et al.*, 1974). These findings suggested that inflammatory reactions produced by x-rays in lung tissues were largely responsible for stimulation of clonogenic growth of tumour cells in the organ.

In this paper we describe experiments in which tumour CFE was measured in rats which were injected with Compound 48/80, cellulose sulphate (CS) and with a preparation of normal rat lung homogenate (LH), which are agents that induce inflammatory reactions in tissues by releasing autacoids, including histamine, 5-hydroxytryptamine, bradykinin and prostaglandins, which act as pharmacological mediators of inflammation (Brocklehurst, 1971; Di Rosa, Giroud and Willoughby, 1971).

### MATERIALS AND METHODS

Female Caworth Farm strain (SPF) rats were injected intravenously with single cell suspensions of Walker (W-256) or Yoshida (Y-P388) tumour cells. Lung tumour macrocolonies were counted on the surfaces of the lungs 7–8 days later, as described previously (van den Brenk *et al.*, 1973*a*). The number of macrocolonies ($N_L$) produced in the lungs by the intravenous injection of $N$ tumour cells was counted in each rat to determine CFE.

### *Supply and preparation of mediators of inflammation and other agents*

*Cellulose sulphate* (*CS*).—Synthetic polysaccharide sulphuric acids are potent anticoagulants referred to as "synthetic heparins" (Bergstrom, 1935). They also cause extensive plasma kininogen depletion with the release of bradykinin in the rat (Rothschild, 1968). Cellulose sulphate was prepared from Whatman cellulose powder CC41 in microgranular form (W. & R. Balston Ltd) using pyridine and chorosulphonic acid, according to the method of Astrup, Galsmar and Volkert (1944); 2·5 g of the powder yielded 9·1 g CS. CS was dissolved in distilled water for intravenous injection of rats.

*Compound* 48/80.—This substance is a condensation product of p-methoxyphenethyl-methylamine with formaldehyde; injected into the mammal Compound 48/80 liberates tissue histamine (Paton, 1951) and also 5-hydroxytryptamine, another mediator of inflammation (Lewis, 1958). Compound 48/80 (Batch 46664) was kindly donated by Wellcome Research Laboratories, Kent; it was dissolved in distilled water for intraperitoneal or subcutaneous injection of rats.

*Other agents.*—Other compounds used for injection of animals were histamine phosphate and 5-hydroxytryptamine creatinine sulphate (BDH Ltd) and dexamethasone sodium phosphate (Decadron; Merck, Sharp & Dohme Ltd). Bradykinin (BRS 640) was kindly donated by Sandoz Ltd and preservative-free mucous heparin was supplied by Weddell Laboratories. Prostaglandins $PGE_1$ and $PGF_{2\alpha}$, generously made available by Miss S. Taylor, Department of Pharmacology, St Thomas' Hospital, were prepared in alcoholic carbonate solution (pH 6–7·5) for intravenous injections.

*Rat tissue homogenates.*—Six- to 8-week old rats were deeply anaesthetized with pentobarbitone sodium and exsanguinated. The lungs were removed, washed in saline and weighed. They were then minced in ice-cold isotonic saline (1 g lung in 2 ml saline), homogenized and centrifuged at 2000 *g*. An aliquot of the supernatant was used to determine the total protein concentration using Folin's method (Lowry *et al.*, 1951). The remaining supernatant was diluted with isotonic saline for intravenous or subcutaneous injection of rats.

### *Biological and toxicity tests*

(i) *Blood: clotting and leucocyte counts.*—Rats were anaesthetized with 35 mg pentobarbitone sodium (Nembutal) per kg body weight injected intraperitoneally, and 3 ml ventricular heart blood was withdrawn into a standard sized test tube. The clotting time was determined at 21°C. The normal range was 45–125 s under the conditions of the test. Leucocyte counts were made in venous blood using a Neubauer counting chamber.

(ii) *Rat paw inflammation.*—The inflammatory effects produced by Compound 48/80 and CS were determined by injecting 0·1 ml of the solution of either agent into an interdigital space of one hind paw and 0·1 ml distilled water into the contralateral paw of lightly anaesthetized rats. Two observers independently recorded changes in the colour, surface temperature and swelling of the paws at 5–15 min intervals for 1 h after injection. The maximum inflammatory reaction produced in each paw was scored semi-quantitatively as: no effect (0), definite reddening, warmth and swelling (+) or red, hot and marked swelling (++). Two rats were used for each dose of the compound tested.

(iii) *Toxicity and lethality.*—Groups of rats were injected intravenously with CS or preparations of lung homogenate in graded amounts to determine the doses required to kill 50% of rats ($LD_{50}$). Half of the $LD_{50}$ dose for each agent was designated the maximum tolerated dose (MTD). MTD for Compound 48/80 was based on the findings of Feldberg and Talesnik (1953) and on previous experience in the use of this agent in rats. Prostaglandins ($PGE_1$ and $PGF_{2\alpha}$) were injected intravenously in doses of 1 mg/kg body weight. Both agents produced shock, tachypnoea and dyspnoea, accompanied by vasodilatation ($PGE_1$) or vasoconstriction ($PGF_{2\alpha}$) of the skin, but the rats appeared to have recovered completely 1 h later. Insufficient amounts of prostaglandin were available to estimate $LD_{50}$ and MTD dosages.

*Adrenalectomy*

Bilateral total adrenalectomy ($TA_X$) or bilateral medullary adrenalectomy ($MA_X$) were performed under pentobarbitone anaesthesia through a midline dorsal approach 2 days before the rats were injected with tumour cells. Rats subjected to $TA_X$ were given daily subcutaneous injections of 1 mg cortisone acetate, their diet was supplemented with dried peas and their drinking water was replaced with 1% sodium chloride.

*Incubation of W-256 cells with CS or LH*

Freshly harvested W-256 ascites fluid was added to medium 199 (containing 10% horse serum). The final tumour cell concentration was $10^6$ cells/ml. One 5 ml aliquot was placed in a culture flask, gassed with 95% $O_2$/5% $CO_2$ and incubated at 36°C for 30 min; 1 mg CS dissolved in 0·1 ml normal saline was added to a second 5 ml aliquot before gassing and incubation. The cells from each flask were then washed twice in ice-cold Tyrode solution. Cell viability was based on the nigrosin exclusion test; this showed that $<1$% of the washed cells from control and CS treated cultures were stained. The cultures were then diluted with ice-cold Tyrode solution and $10^4$ cells contained in 0·5 ml from each culture were injected intravenously in 2 groups of 6 rats. Lung colonies were counted 7 days later. Similarly, W-256 cells were incubated with LH (1 part undiluted LH added to 4 ml of medium v/v) or with histamine, 5-hydroxytryptamine or bradykinin ($10^{-4}$ g drug/ml medium final concentration); the incubated cells were washed in Tyrode solution, injected intravenously in rats and assayed for colony formation in the lungs of rats.

RESULTS

*Pharmacological effects of agents used to induce inflammation*

(i) *Local tissue reactions.*—Interdigital injections of 1 $\mu$g of Compound 48/80 caused a definite (+) inflammatory reaction in rats. The injected paw became red, warm and swollen within 1–2 min; some swelling remained 1 h later. Larger doses (10–100 $\mu$g) 48/80 caused more marked (++) reactions. Interdigital injections of 10–1000 $\mu$g CS caused (+) to (++) inflammatory reactions which took somewhat longer (5–10 min) to develop than after 48/80. Very marked inflammation of the paw was produced by an interdigital injection of 0·1 ml LH (diluted 1 in 16 v/v). The swelling lasted for 2–3 h but had subsided after 24 h. LH preparations were slightly acidic; neutralization to pH 7–7·4 did not alter the inflammatory response, but injection of LH heated at 60°C for 30 min caused less swelling of the paw.

(ii) *Systemic effects and toxicity.*—Compound 48/80 injected intraperitoneally in doses of 100 $\mu$g or more causes mast cell rupture, accompanied by the release of biogenic amines—particularly histamine and 5-hydroxytryptamine (Lewis, 1958); depletion is followed by resynthesis of the autacoids in the tissues (Paton, 1951; Riley and West, 1955). Compound 48/80 caused hypotension, pulmonary oedema and an anaphylactic shock-like state attributed principally to the release of tissue histamine; rats injected with larger doses (500–1000 $\mu$g) 48/80 died from haemorrhagic pulmonary oedema. Rats injected with 5–10 mg CS/kg body weight rapidly showed signs of shock and dyspnoea but survived; rats injected with $>10$ mg CS/kg body weight died from pulmonary haemorrhage and oedema. CS does not release histamine but depletes plasma kininogen and causes the release of the peptide bradykinin, a mediator of inflammation which is also largely responsible for the toxic effects of intravenously injected CS; CS strongly inhibits clotting of the blood and increases the fibrinolytic activity of the blood (Rothschild, 1968). We found that $PGE_1$ and $PGF_{2\alpha}$ and bradykinin had no significant effects on blood clotting time. Compound 48/80 slightly shortened clotting time in the rat. CS added to rat blood *in vitro* prolonged clotting time, which was similarly increased in the blood taken from rats which had been injected with CS (Fig. 1). The fibrinolytic effect of CS is most marked in rats injected with 1–3 mg CS/kg body weight; further increase in dose to 10 mg CS/kg causes fibrinolytic activity to decrease to normal, whereas the anticoagulant effect of the agent continues to increase with increase in dose (Rothschild, 1968, Fig. 1). Neither CS nor Compound 48/80 produces significant changes in haematocrit levels, blood platelets or plasma protein (Rothschild, 1968). However, both agents caused leucocyte counts to increase by 30–100% within 15 min after injection.

The maximum tolerated dose (MTD) of LH injected intravenously in rats was 0·4 ml of diluted LH (1 part LH : 32 parts distilled water v/v). This dose caused shock and respiratory difficulty, from which rats rapidly recovered, but did not alter blood clotting time. The toxic effects of larger doses of LH were similar to those produced by CS, *i.e.* shock, cyanosis and respiratory difficulty, except that LH caused rapid loss of consciousness which was not preceded by convulsions. Death often occurred before there was evidence of pulmonary oedema and haemorrhage and appeared to be due to cardiac arrest, but these physiological changes have not been investigated. LH caused rupture of mast cells in the mesenteries and subcutis of the rat. The local and systemic effects of LH resembled those of Compound 48/80 and CS in the rat, and it is assumed that the common mechanism of action of the agents is the release of mediators of inflammation from the tissues of the animal.

FIG. 1.—Effect of adding cellulose sulphate (CS) to rat blood *in vitro* (closed symbols), or of intravenously injecting rats with a single dose CS 10 min before bleeding (open symbols), on clotting time of blood.

*Effects of CS, Compound 48/80 and LH on CFE*

A single intravenous injection of 10 mg CS/kg body weight given shortly before or after an intravenous injection of 5 $\times 10^3$ W-256 tumour cells caused marked increases in CFE in the lungs of 25-day old rats (Table I). The greatest increase in CFE (accompanied by $\sim$100% increase in lung weight due to tumour growth) occurred when CS was injected 10 min before the tumour cells. Stimulation of CFE was decreased when CS was injected 2 h before or after the tumour cells, but CFE was significantly raised even when the cells were injected 24 h after CS. CS had no significant effect on CFE when it was injected 24 h after the tumour cells. Consequently, the degree of stimulation of CFE by CS appeared to depend on the presence and intensity of the physiological reaction (inflammation) induced in the rat at the time of injection and implantation of the tumour cells, or in the first few hours after seeding in the tissues; as the inflammatory reaction resolved, CFE decreased *pari passu*. Compound 48/80 injected, intraperitoneally in maximum tolerated dosage, also enhanced CFE but to a lesser extent than CS. An intravenous injection of LH also stimulated CFE (Table II). CS and Compound 48/80 also stimulated CFE in rats which were 6 weeks old (Table I, Fig. 2). Between 4 and 6 weeks of age, CFE of tumour cells in the lungs and other organs of the rat has been shown to decrease markedly, even if assays are performed in rats given sublethal whole body irradiation to suppress immunity (van den Brenk *et al.*, 1973*a*). Stimulation of CFE by CS was dose-dependent (Fig. 3). The dose–effect relationship for CS on CFE correlates with the effect of CS of inhibiting haemocoagulation (Fig. 1, 3) but not with its effect on fibrinolytic activity (see above).

*Effects of pharmacological mediators of inflammation (biogenic amines, bradykinin and prostaglandins) on CFE*

Previous preliminary studies had

TABLE I.—*Effect on CFE in Lungs of Female Rats Injected Intravenously with W-256 Tumour Cells of: (i) a Single Dose of 10 mg Cellulose Sulphate (CS) per kg Body Weight Injected Intravenously 10 min–24 h Before or After the Injection of Tumour Cells, (ii) 2 Doses of 1 mg Compound 48/80 per kg Body Weight Injected Intraperitoneally 10 min Before and 3 h After the Injection of Tumour Cells. Weanling (25-day old) Rats were Injected with 5 $\times 10^3$ W-256 Cells and 6-week Old Rats with $10^4$ W-256 Cells. Eight Rats Per Group ($W_1$ Mean Body Weight on Day − 1 and $W_2$ Mean Body Weight when Rats were Killed on Day + 7; Tumour Cells Injected on Day 0)*

| Treatment | $W_1$ (g) | $W_2$ (g) | $N_L$ | Organ weight (g) Lungs | Spleen | Thymus |
|---|---|---|---|---|---|---|
| 25-day old rats | | | | | | |
| I. Nil | 64 | 108 | 33±9 | 0·84±0·02 | 0·59±0·02 | 0·38±0·03 |
| II. CS (−24 h) | 62 | 104 | 75±12 | 0·87±0·02 | 0·57±0·02 | 0·37±0·02 |
| III. CS (−2 h) | 61 | 103 | 310±28 | 1·06±0·06 | 0·58±0·03 | 0·43±0·02 |
| IV. CS (−10 min) | 63 | 103 | >500* | 1·68±0·15 | 0·55±0·02 | 0·41±0·02 |
| V. CS (+2 h) | 63 | 103 | 123±26 | 0·96±0·04 | 0·54±0·03 | 0·37±0·02 |
| VI. CS (+24 h) | 60 | 103 | 26±14 | 0·84±0·04 | 0·59±0·03 | 0·40±0·02 |
| VII. Compound 48/80 (−10 min, +3 h) | 65 | 106 | 125±22 | 0·92±0·04 | 0·59±0·02 | 0·41±0·02 |
| Six-week old rats | | | | | | |
| VIII. Nil | 119 | 154 | 15±6 | 1·09±0·03 | 0·69±0·03 | 0·43±0·02 |
| IX. CS (−10 min) | 111 | 141 | >300* | 1·60±0·15 | 0·62±0·04 | 0·49±0·02 |
| X. Compound 48/80 (−10 min, +3 h) | 113 | 142 | 137±43 | 1·22±0·16 | 0·66±0·04 | 0·41±0·02 |

* Estimates; colonies confluent in many parts of lungs; blood stained pleural effusions containing $10^6$–$10^7$ tumour cells per ml were present in all rats in groups IV and IX, and in 3 rats in each of groups III, VII and X.

TABLE II.—*Effect on CFE in the Lungs of* 0·4 *ml Lung Homogenate Supernatant* (*LH*)* *Diluted* 1 *in* 32 *with Distilled Water* (*v/v*), *Injected Intravenously in* 6-*week Old Rats* 10 *min Before the Intravenous injection of* $5 \times 10^3$ *W*-256 *Tumour Cells. Tumour Macrocolonies were Counted* 8 *Days After the Tumour Cells were Injected* (6 *Rats Per Group; Controls Injected Intravenously with* 0·4 *ml Distilled Water in Place of LH*); $W_1$, $W_2$ *Initial and Final Body Weights*

| Group | Mean body weight (g) W₁ | Mean body weight (g) W₂ | Number of lung colonies $N_L$ (range) | Organ weights (g) Lungs | Organ weights (g) Spleen | Organ weights (g) Thymus |
|---|---|---|---|---|---|---|
| Control | 116±3 | 147±3 | 15±1 (11–18) | 0·98±0·02 | 0·69±0·02 | 0·43±0·01 |
| LH | 117±4 | 146±5 | 65±17 (20–136) | 1·03±0·03 | 0·68±0·04 | 0·47±0·05 |

* Undiluted LH contained 33·5 mg protein per ml.

TABLE III.—*Effects on CFE in Lungs of Rats of* (*A*) *Histamine Phosphate,* 5-*hydroxytryptamine and Bradykinin, Injected Intravenously and Simultaneously with* $5 \times 10^2$ *W*-256 *Cells, and* (*B*) *Prostaglandins* $PGE_1$ *and* $PGF_{2\alpha}$, *Similarly Added to Intravenously Injected* $10^4$ *Y-P*388 *Cells. Compounds were Added to the Cells Immediately Before Injection. Mean Body Weights of Rats* (6–8 *Rats per Group*) *were* $W_1$ *at Time of Injection and* $W_2$ 7 *Days Later when Rats were Killed to Count Tumour Colonies*

| Added compound(s) (dose per rat) | $W_1$ (g) | $W_2$ (g) | Number of lung colonies ($N_L$) |
|---|---|---|---|
| A. I. Nil | 80 | 120 | 4±1 |
| II. 1 mg histamine | 84 | 123 | 5±2 |
| III. 0·5 mg 5-hydroxytryptamine | 74 | 112 | 8±4 |
| IV. 1 μg bradykinin | 73 | 114 | 6±2 |
| V. 1 μg bradykinin *plus* 1 mg histamine | 74 | 113 | 5±1 |
| VI. 1 μg bradykinin 1 mg histamine 0·5 mg 5-hydroxytryptamine | 86 | 118 | 9±4 |
| B. I. Nil (solvent)* | 108 | 137 | 17±6 |
| II. 100 μg $PGE_1$ | 98 | 130 | 24±9 |
| III. 100 μg $PGF_{2\alpha}$ | 98 | 128 | 25±7 |

There were no significant differences in gain in body weight ($W_2$–$W_1$), $N_L$ and in final weights of spleen, thymus and lungs between control and treated rats in experiments A and B.

* An equal volume of alcoholic-carbonate buffer that was used to dissolve prostaglandins for addition to injected cells in II and III was added to cells injected in I.

shown that CFE was not significantly affected by intravenous or intraperitoneal injection of rats with histamine, 5-hydroxytryptamine or adrenergic amines (noradrenaline, adrenaline, isopropylnoradrenaline or methoxamine) in doses of one quarter to one half the $LD_{50}$ level, given 5–10 min before the injection of the tumour cells (unpublished data). Neither did CFE change when Compound 48/80 was administered daily for 3–10 days to deplete the tissue amines to less than 10% of normal values before the injection of tumour cells (van den Brenk *et al.*, 1973*b*). Also, the treatment of rats with antihistaminic drugs had no effect on CFE (van den Brenk *et al.*, 1974). Table III shows the results of a further experiment in which the autacoids liberated by 48/80 and CS, namely histamine, 5-hydroxytryptamine and bradykinin were injected intravenously, either singly or in combination, together with W-256 tumour cells. These treatments had no significant effects on CFE. The prostaglandins, $PGE_1$ and $PGF_{2\alpha}$, added in doses of 100 μg to the tumour cells immediately before intravenous injection, also failed to stimulate CFE. This dosage in rats was approximately 1 mg PG/kg body weight. The injection of $PGE_1$ and $PGF_{2\alpha}$ added to the tumour cells caused shock accompanied by respiratory and vasomotor effects which did not

FIG. 3.—Number of tumour macrocolonies produced in lungs of 8-week old rats injected with W-256 cells 10 min after a single dose of cellulose sulphate had been injected intravenously. Eight rats per point; $5 \times 10^3$ W-256 cells (closed symbols) and $10^3$ cells (open symbols) were injected intravenously and colonies counted 8 days later.

differ from those produced by injection of the drugs alone in the same dosage.

Consequently, stimulation of CFE by Compound 48/80 and CS, and probably by tissue homogenate as well, does not appear to be due directly to the individual pharmacological effects of the autacoids which are liberated in tissues and act as mediators of inflammation (Brocklehurst, 1971; Di Rosa, Giraud and Willoughby, 1971), but rather to some other component(s) of the inflammatory reaction.

*Effects of CS on CFE in tumour immunized rats*

In our experience, the most efficient method of immunizing the rat against growth of allogeneic tumour cells is by injecting intact tumour cells into the muscle of the leg of the rat so that solid tumour (1–2 g in weight) develops after 7–10 days growth, when the rat's immunity to growth of a second challenge of the same tumour is greatly increased. The $TD_{50}$ value of a second challenge of W-256 cells injected into the muscle of the opposite leg was $> 10^6$ cells compared with $< 10$ cells (primary challenge), and for intravenously injected W-256 cells immunization reduced CFE in the lungs $< 0{\cdot}0001$ (van den Brenk *et al.*, 1973*a*). This method of immunization was based on that of Haddow and Alexander (1964) for growth of immunogenic methylcholanthrene induced sarcomata in the rat. These workers found that the immunity produced in the rat by a growing tumour was much greater than that produced by repeated injections of large numbers ($10^6$–$10^7$) of heavily (lethally) irradiated (HR) cells over a period of time. Table IV shows that in rats immunized by the growth of a primary challenge of W-256 cells in leg muscle, CS did not reverse the effects of immunity on the growth of a secondary challenge—a finding which parallels that obtained when local thoracic irradiation was used to stimulate CFE of allogeneic tumour cells in the lungs of rats (van den Brenk *et al.*, 1973*b*). The fact that CS increased CFE by a factor of $\sim 15$ in unimmunized and in immunized rats alike is taken to reflect a competition between the inflammatory state induced by CS and that of immunity on tumour growth; each mechanism would appear to act independently on survival and growth of tumour cells. It is significant that while inflammation did not directly antagonize the action of tumour immunity, it protected a proportion of newly seeded tumour cells from immunodestruction for the time needed for these cells to clone and thus increase CFE. Marked splenic enlargement, which is produced in immunized rats by the growth of tumour, was not affected by CS, neither did immunization or CS significantly affect the weight of the thymus (Table IV).

TABLE IV.—*Effect on CFE in Lungs of Rats of* 10 *mg CS per kg Body Weight Injected Intravenously* (*IVI*) 10 *min Before the* Primary *Challenge of* $10^4$ *W*-256 *Tumour Cells* (*IVI*) *in Unimmunized Rats* (*A and C*) *or Before the* Secondary *Challenge of* $10^4$ *W*-256 *Cells* (*IVI*) *in Rats which Had Been Immunized Against This Tumour by Allowing an Intramuscular Injection of* $10^4$ *W*-256 *Cells to Grow for* 7 *Days Into a Solid Tumour in the Right Leg* (*B*). *The Unimmunized Rats Used in* (*C*) *were Injected Intramuscularly with* 2 *mg Dexamethasone* 2 *h before* 10 *mg CS per kg Body Weight or an Equal Volume of Distilled Water was Injected IVI, Followed by* $10^4$ *W*-256 *Cells IVI* 10 *min Later; Control Rats in Subgroups in A and B not Injected with CS Also Received Equal Volumes of Distilled Water IVI;* (6–8 *Rats Per Group*)

| Group | Treatment with CS | $W_1$* (g) | $W_2$* (g) | $N_L$ | Weight of organs (g) Lungs | Spleen | Thymus |
|---|---|---|---|---|---|---|---|
| A | – | 84 | 150 | 26±9 | 1·02 | 0·70 | 0·50 |
| | | | | (6–64) | ±0·01 | ±0·03 | ±0·05 |
| Not immunized | + | 88 | 146 | >300 | 1·31 | 0·66 | 0·50 |
| | | | | | ±0·06 | ±0·02 | ±0·03 |
| B | – | 87 | 156 | 1±0·07 | 1·08 | 1·08 | 0·46 |
| | | | | (0–3) | ±0·03 | ±0·09 | ±0·03 |
| Immunized† | + | 85 | 143 | 15±6 | 1·12 | 1·15 | 0·44 |
| | | | | (3–46) | ±0·01 | ±0·17 | ±0·03 |
| C | – | 87 | 125 | 15±5 | 0·93 | 0·60 | 0·15 |
| | | | | (2–27) | ±0·02 | ±0·07 | ±0·03 |
| Not immunized; Dexamethasone | + | 94 | 135 | 164±28 | 0·99 | 0·55 | 0·12 |
| | | | | (64–250) | ±0·03 | ±0·03 | ±0·01 |

* $W_1$ mean body weight on Day −7 when rats in group B were immunized by injecting W-256 cells intramuscularly; $W_2$ mean body weight on Day +8 when rats were killed (8 days after $10^4$ W-256 cells IVI on Day 0). Values for $W_2$ group C rats were significantly lower due to decreased growth of rats following the injection of the steroid on Day 0.

† All rats in both subgroups in B had developed large solid tumours in the right leg when killed on Day +8 weighing 2–5 g, and small (< 1 g) to large (> 2 g) metastases in pelvic lymph nodes; there was no significant difference between the 2 subgroups with respect to growth of the primary tumours or of lymph node metastases.

TABLE V.—*Effect of* 8 *mg Cellulose Sulphate per kg Body Weight IVI* 10 *min Before* $10^4$ *Y-P*388 *Cells IVI on Number of Lung Tumour Colonies* ($N_L$) *Produced* 8 *Days Later in* 7*-week Old Rats in which Bilateral Total Adrenalectomy* ($TA_x$) *or Medullary Adrenalectomy* ($MA_x$) *had been Performed* 2 *Days Preceding the Injection of Tumour Cells.* $W_1$ *and* $W_2$ *are Mean Body Weights on the Day of Operation and* 10 *Days Later when Rats were Killed Respectively;* 6–8 *Rats Per Group*

| Group (treatment) | $W_1$ (g) | $W_2$ (g) | $N_L$ | Organ weight (g) Lungs | Spleen | Thymus |
|---|---|---|---|---|---|---|
| I. Nil* | 157±5 | 177±4 | 9±4 | 1·08±0·02 | 0·79±0·05 | 0·43±0·01 |
| II. $MA_x$ | 158±3 | 187±5 | 5±3 | 1·22±0·03 | 0·89±0·04 | 0·55±0·04 |
| III. $TA_x$ | 150±4 | 157±4 | 3±2 | 1·00±0·01 | 0·69±0·04 | 0·38±0·02 |
| IV. CS* | 156±4 | 175±5 | 20±2 | 1·08±0·03 | 0·75±0·06 | 0·41±0·04 |
| V. $MA_x$ *plus* CS | 156±6 | 180±8 | 25±7 | 1·25±0·06 | 1·02±0·06 | 0·50±0·06 |
| IV. $TA_x$ *plus* CS | 154±4 | 163±5 | 27±3 | 1·02±0·04 | 0·77±0·03 | 0·39±0·02 |

* Anaesthetic only on the day adrenalectomies were performed in rats in other groups.

*CFE in adrenalectomized rats*

CFE of W-256 cells in the lungs of totally adrenalectomized rats was slightly reduced ($P < 0{\cdot}05$). This reduction in CFE may be related to inhibition of rate of body growth after total adrenalectomy (Table V). Bilateral medullary adrenalectomy had less effect on both body growth and on tumour CFE. Neither total nor medullary adrenalectomy prevented stimulation of CFE by CS. This suggests that the effect of CS on CFE is not due to the stress syndrome (Selye, 1950) associated with the systemic release of hormones (including adrenaline) from the adrenals.

*Reduction of effect of CS on CFE by dexamethasone*

A large single dose of 2 mg dexamethasone, an anti-inflammatory steroid, injected intramuscularly 2 h before the injection of CS, markedly reduced the effect of CS on CFE (Table IV).

*Assays of W-256 cells incubated with CS, LH and mediators of inflammation*

W-256 cells were incubated with 0·2 mg CS per ml of medium. This concentration of CS ($2 \times 10^{-4}$ g per ml) was chosen as approximately equal to the mean concentration of CS produced in the blood of rats by the intravenous injection of the maximum tolerated dose of 10 mg CS per kg body weight, assuming that the agent is distributed uniformly in the blood and the blood volume of the rat is approximately 50 ml blood per kg body weight. Rats injected intravenously with $10^4$ W-256 cells incubated with CS developed $27 \pm 7$ lung colonies, compared with $45 \pm 27$ colonies in rats injected with $10^4$ cells which had been incubated without CS. It is concluded that CS had no direct effect on the survival or clonogenicity of W-256 cells. Similarly, CFE of W-256 cells incubated with LH or with the mediators histamine, 5-hydroxytryptamine and bradykinin, was not significantly different from that of cells incubated without the addition of these agents (results not tabulated). The final concentration ($10^{-4}$ g per ml) of the mediators present in the incubation medium greatly exceeded the local tissue concentrations required to induce inflammatory oedema in rats, and the maximum doses tolerated by rats (measured as dose per unit body weight) when the drugs were administered parenterally. The mediators tested *in vitro* on sensitive preparations of contractile rat and guinea-pig tissues (including granulation tissue) induced maximum contractile responses at a concentration of less than $10^{-4}$ g per ml in the test bath (unpublished data; Gaddum, 1949; Majno *et al.*, 1971).

*Effect of anticoagulant treatment with heparin on CFE*

Anticoagulation treatment of rats with preservative-free heparin had no significant effect on CFE (Table VI), even if the rats had been given local thoracic irradiation 7 days before the cells were injected to increase CFE as described previously (van den Brenk *et al.*, 1973*b*). Similarly, CFE was not affected by intraperitoneal injection of rats with 250 i.u. heparin 10 min before the tumour cells were injected intravenously; the results of this experiment were essentially similar to those shown in Table V and are not tabulated.

TABLE VI.—*Effect on CFE in Lungs of Rats of 250 i.u. Preservative-Free Heparin (0·05 ml) Added to W-256 Cells (Suspended in 0·45 ml) and Injected Intravenously. Weanling Female Rats (6 Per Subgroup) were given 1000 rad Local Thoracic Irradiation* Under Anaesthesia 7 Days before the Injection of Tumour Cells in Groups B, C and D to Increase CFE; the Rats in Group A were not Irradiated. Heparin was Added to the Injected Cell Suspension in the 4 Subgroups Marked (II) Only*

| Group | $N$ Number of cells injected | $N_L$ Number of lung colonies |
|---|---|---|
| A (I) | $2\times10^3$ | $16\pm3$ |
| (II) | $2\times10^3$ | $26\pm7$ |
| B (I) | $10^2$ | $30\pm5$ |
| (II) | $10^2$ | $45\pm8$ |
| C (I) | $5\times10^2$ | $106\pm16$ |
| (II) | $5\times10^2$ | $105\pm20$ |
| D (I) | $10^3$ | $161\pm15$ |
| (II) | $10^3$ | $183\pm25$ |

* X-radiation technique has been described previously (van den Brenk *et al.*, 1973*b*).

However, it was found that when 250 i.u. heparin containing 0·15% chlorocresol as preservative was added to the tumour cells, CFE was markedly reduced. On the other hand, CFE was not significantly affected by the same dose of heparin (containing the preservative) if it was injected intraperitoneally 10 min before the intravenous injection of tumour cells. Lung colony assays performed with tumour cells which had been incubated with $10^{-6}$ concentrations of chlorocresol for 30 min showed that this substance is highly toxic to tumour cells and reduced CFE (unpublished data). The toxic effects of the preservative employed in preparing most brands of heparin used in medicine may account for some experimental findings in which treatment with this anticoagulant was found to cause modest reductions in growth of injected tumour cells and in the development of metastases.

*Subcutaneous growth of tumour in inflamed paw*

Inflammation and swelling of the hind paw of the rat were produced by the interdigital injection of either 0·1 ml of diluted (1 : 4 v/v) LH, or 100 μg CS dissolved in 0·1 ml distilled water. Ten min later 10 W-256 cells suspended in 0·1 ml Tyrode solution were injected into the swollen paw and also into the contralateral (untreated) paw. In a group of 6 rats injected with LH palpable, actively growing, haemorrhagic tumours developed in the treated paws of 5 rats and no tumours in untreated paws. The 5 tumours were 2–10 mm in diameter at 21 days and continued to increase in size for a further 7 days when the rats were sacrificed. In the 6 rats treated with CS similar sized tumours grew in 4 treated paws, and in one rat a small tumour (2 mm in diameter) had developed in the untreated paw but was regressing. In similar groups of rats injected with 33 or 100 W-256 cells tumours developed in both the treated (CS or LH injected) and untreated paws of all rats, but larger tumours were present in the treated paws of most rats, particularly in the group injected with LH. In these experiments the injection of tumour cells into the swollen paw did not significantly affect the rate of resolution of the inflammatory reactions; there was no swelling of either paw 24 h after the injections.

DISCUSSION

Cellular damage and death caused by physical or chemical injury in the organism evokes local vascular and other physiological reactions at the site of injury, accompanied by certain systemic reactions which comprise inflammation. It is now widely accepted that the inflammatory reaction is mediated by the release of certain autacoids—substances present in tissues which have been isolated and chemically identified, and cause pharmacological reactions of a typically inflammatory nature (Di Rosa *et al.*, 1971). The most important known mediators of inflammation are the biogenic amines, histamine and 5-hydroxytryptamine, the peptide bradykinin and a group of cyclic oxygenated $C_{20}$ fatty acids—the prostaglandins (Brocklehurst, 1971); other pharmacologically active substances have been extracted from tissues, such as SRS (" slow reacting substance "), but their role in inflammation is less certain.

Since inflammation produced by tissue damage is the prelude to regeneration and repair, which involves blastogenesis and proliferative cell growth, it is reasonable to suppose that acute inflammatory exudates not only facilitate the growth of normal tissue (repair) but might act similarly with respect to tumour cells owing to the presence of growth promoting substances (GSS). This supposition prompted the experiments in which the effects of inflammatory reactions induced with Compound 48/80 and CS on survival and clonogenic growth of tumour cells in the rat were measured. These agents were chosen because they cause cellular injuries of a peculiar type, which release biogenic amines and bradykinin from tissues and cause local and systemic reactions characteristic of the inflammatory response. Since inflammation is attributed to the biochemical action of products of cell injury *in vivo*, the effects of injecting rats with a preparation of homogenized rat lung (LH) on tumour growth was tested, since LH injected subcutaneously induced a marked inflammatory reaction. We have shown that parenteral administration of Compound 48/80, CS or LH greatly enhanced survival and clonogenic growth in the lungs of the rat of intravenously injected allogeneic tumour cells prepared in single cell suspension. The growth of a small number of subcutaneously injected tumour cells was similarly enhanced if the cells were injected into inflamed tissue. We have previously shown that local x-irradiation increased CFE of allogeneic tumour cells in the lungs (van den Brenk *et al.*, 1973*a*) and in the liver and kidneys of the rat (van den Brenk and Kelly, 1973*c*). The principal effect of x-rays on tissues is inhibition of proliferative cell growth; this results in cell death which stimulates inflammation. We consider this inflammatory state to be the principal cause of stimulation of tumour growth in tissues damaged by x-rays and other forms of injury. The fact that treatment of rats with anti-inflammatory steroids decreased stimulation of tumour CFE produced by x-rays (van den Brenk *et al.*, 1974) and by CS (Table I) supports this view.

The pharmacological changes induced in rats by the injection of single large doses of the individual mediators of inflammation, histamine, 5-hydroxytryptamine, bradykinin and prostaglandins $PGE_1$ and $PGF_{2\alpha}$, did not result in stimulation of CFE. This suggests that the growth promoting effects of inflammation depend on an integration of the individual actions of the various mediators released by tissue injury. However, although both CS and Compound 48/80 stimulate CFE, CS predominantly releases bradykinin, whereas Compound 48/80 releases biogenic amines. This suggests that some other agent is released from injured tissues, or is contained in inflammatory exudates, which promotes growth either alone or by complementing the action of the mediators in this respect. The fact that the incubation of tumour cells with CS, Compound 48/80, LH or pharmacological mediators of the inflammatory response did not affect their viability or alter CFE indicates that the growth promoting activity obtained *in vivo* is associated with the inflammatory exudate. Direct support for this view is provided by the finding that repeated intravenous injections of rats with freshly harvested cell-free tumour ascites plasma stimulated CFE, if large amounts (0·5–1·0 ml) of the plasma were injected intravenously at 60–90 min intervals within 4 h *after* the tumour cells were injected intravenously; heating the ascites plasma at 60°C for 30 min abolished its effect of stimulating CFE (unpublished data).

Many workers have found that treatment of mice and rats with anticoagulants inhibited growth of transplanted tumours and spread of metastases (Wood, Holyoke and Yardley, 1961). Also, it has been suggested that the presence of fibrin and thromboplastin are important in increasing take and growth of transplanted tumours (Grossi, Agostino and Cliffton, 1960; Hewitt, Blake and Porter, 1973). Clotting of blood and laying down of fibrin also provides a support for the growth of regenerating blood vessels (Stearns, 1940*a*, *b*). However, CFE of tumour cells in the lungs was not affected by anticoagulant treatment with mucous heparin, and was greatly stimulated by CS, a synthetic heparin which is a powerful anticoagulant and also increases fibrinolysis (Rothschild, 1968). However, it is conceivable that the envelopment of tumour cells by fibrin of clotted exudates can decrease the rates of loss by diffusion of GSS produced by the tumour or the tumour bed. This effect would seem to be most important in supporting survival and growth of single tumour cells which seed in tissues. Thus, it has been shown that local variations in the fluid micro-environment and diffusion boundary layer of cells greatly affects the dynamics of growth (Stoker, 1973; Dulbecco and Elkington, 1973). It is suggested that the capacity of different types of tumours to produce GSS varies widely and may be a quality closely related to that of malignant (autonomous) behaviour of a tumour and may also affect the chances of survival and clonogenicity of seeded tumour cells. The feeder cell phenomenon *in vitro* (Puck and Marcus, 1956) and the Révész phenomenon *in vivo* (Révész, 1956) have demonstrated that malignant cells produce GSS which greatly affect clonogenic growth. The supplementation of GSS produced by the tumour cell with GSS produced by the tumour bed would increase CFE, particularly if the supply of GSS by host tissues is augmented by inflammatory reactions.

We have shown that inflammation induced in the tissues of the rat did not prevent the actions of tumour–host immunity, established in the same animal, of inhibiting growth of allogeneic tumour cells; the two reactions, inflammation and immunity, appeared to act independently and to compete in determining the net survival of the seeded tumour cells. This finding parallels that previously obtained for CFE of tumour cells seeded in the irradiated lungs of immunized rats (van den Brenk *et al.*, 1973*b*). It was also found previously that the inflammatory reaction induced in rats with Compound 48/80 increased early growth of subcutaneously transplanted xenogenic murine cancer cells (van den Brenk and Upfill, 1958). It follows that stimulation of tumour growth by inflammation may be of much greater consequence when tumour–host immunity is weak or absent, as in spontaneous cancers or grafted syngeneic tumours, than under conditions of competition with the marked immunity produced by transplantation of allogeneic and xenogenic tumours. Since immune reactions result in cell death, which induces inflammation *per se*, a situation can arise wherein the survival and growth of tumour cells are inhibited by immunity but stimulated by inflammation caused by the destruction of participating host and tumour cells. The net result may be " immunological " enhancement of tumour growth. Similarly, inflammatory reactions induced by the injury and death of normal tissues being replaced by growth of solid tumours may contribute to " invasiveness " and progressive growth of solid tumours, even when immunity has developed. Indeed, to a limited extent tumour antigenicity conceivably favours the " take ", growth and spread of the tumour cell.

The mechanism involved in " cocarcinogenesis " appears to be closely related to and perhaps wholly due to a non-specific growth promoting effect of inflammation of tissues caused by " co-carcinogens "; agents which have been defined as physical or chemical agents which " alone are not carcinogens, when applied along with or after the application of carcinogenic agents may increase the carcinogenic effect ", and " precipitate neoplasia in an area of tissue already prepared for it by the previous application of carcinogens " (Willis, 1950). This interpretation of cocarcinogenesis is in agreement with that of Menkin (1961), who prepared a diffusible growth promoting factor from inflammatory exudates induced in rats, which was heat stable and inactivated by ribonuclease and trypsin, and acted as a cocarcinogen in mice and rabbits. Menkin reasoned that the liberation of endogenous GSS by inflammation offered a reasonable explanation for the induction of repair (regenerative growth). He also showed that the growth promoting activity of dialysates prepared from tissues of young (actively growing) animals was more pronounced than that of mature tissues. We consider that the rapid decrease in tumour CFE which occurs with decrease in growth rate of the rat after weaning (van den Brenk *et al.*, 1973*a*) is largely attributable to the same mechanisms, namely, a decrease in tissue GSS. Also, we suggest that the well established effects of injury of stimulating growth and of precipitating the clinical (overt) manifestation of cancer in organs such as breast, bone, testis and skin in man (Willis, 1948) are due primarily to the local growth promoting action of inflammation induced by the injury and do not basically differ from " cocarcinogenesis ".

### REFERENCES

Astrup, T., Galsmar, I. & Volkert, M. (1944) Polysaccharide Sulfuric Acids as Anticoagulants. *Acta physiol. scand.*, **8**, 215.

Bergstrom, S. (1935) Uber die Wirkungsgruppe des Heparins. *Naturwissenschaften*, **23**, 706.

Brocklehurst, W. E. (1971) Role of Kinins and Prostaglandins in Inflammation. *Proc. R. Soc. Med.*, **64**, 4.

Di Rosa, M., Giroud, J. P. & Willoughby, D. A. (1971) Studies of the Mediators of the Acute Inflammatory Response Induced in Rats in Different Sites by Carrageenan and Turpentine. *J. Path.*, **104**, 15.

Dulbecco, R. & Elkington, J. (1973) Conditions Limiting Multiplication of Fibroblastic and Epithelial Cells in Dense Cultures. *Nature, Lond.*, **246**, 197.

Feldberg, W. & Talesnik, J. (1953) Reduction of Tissue Histamine by Compound 48/80. *J. Physiol.*, **120**, 550.

Gaddum, J. H. (1949) *Pharmacology*. Third Ed. London: Oxford University Press.

Grossi, C. E., Agostino, D. & Cliffton, E. E. (1960) The Effect of Human Fibrinolysin on Pulmonary Metastases of Walker 256 Carcinoma. *Cancer Res.*, **20**, 605.

Haddow, A. & Alexander, P. (1964) An Immunological Method of Increasing the Sensitivity of Primary Sarcomas to Local Irradiation with X-Rays. *Lancet*, i, 452.

Hewitt, H. E., Blake, E. & Porter, E. H. (1973) The Effect of Lethally Irradiated Cells on the Transplantability of Murine Tumours. *Br. J. Cancer*, **28**, 123.

Lewis, G. P. (1958) 5-hydroxytryptamine in the Mast Cell of the Rat. In *5-hydroxytryptamine*. Ed. G. P. Lewis. London: Pergamon Press.

Lowry, O. H., Rosebrough, N. J., Farr, A. L. & Randall, R. J. (1951) Protein Measurements with the Folin Phenol Reagent. *J. Biochem.*, **193**, 265.

Majno, G., Gabbiani, G., Horschel, B. J., Ryan, G. B. & Stratkov, P. R. (1971) Contraction of Granulation Tissue *in vitro*: Similarity to Smooth Muscle. *Science, N.Y.*, **173**, 548.

Menkin, V. (1961) Studies of the Growth Promoting Factor of Exudates in Reference: (a) to its Effect on the Development of Spontaneous Neoplasms in Tumour Susceptible Mice; (b) to its Presence in Rabbit Exudate; (c) in its Comparative Growth Potential in Organs of Immature and Adult Animals. *Path. Biol.*, **9**, 861.

Paton, W. D. M. (1951) Compound 48/80: A Potent Histamine Liberator. *Br. J. Pharmac.*, **6**, 499.

Puck, T. T. & Marcus, P. I. (1956) Action of X-Rays on Mammalian Cells. *J. exp. Med.*, **103**, 653.

Révész, L. (1956) The Effect of Tumour Cells Killed by X-rays upon the Growth of Admixed Viable Cells. *Nature, Lond.*, **178**, 1391.

Riley, J. F. & West, G. B. (1955) Tissue Mast Cells; Studies with Histamine-Liberator of Low Toxicity (Compound 48/80). *J. Path. Bact.*, **69**, 269.

Rothschild, A. M. (1968) Some Pharmacodynamic Properties of Cellulose Sulphate, a Kininogen Depleting Agent in the Rat. *Br. J. Pharmac. Chemother.*, **33**, 501.

Selye, H. (1950) Stress. The Physiology and Pathology of Exposure to Stress. *Acta Inc.*, Montreal.

Stearns, M. L. (1940*a*) Studies on the Development of Connective Tissue in Transparent Chambers in the Rabbit's Ear. I 1, 2. *Am. J. Anat.*, **66**, 133.

Stearns, M. L. (1940*b*) Studies on the Development of Connective Tissue in Transparent Chambers in the Rabbit's Ear. II 1. *Am. J. Anat.*, **67**, 55.

Stoker, M. G. P. (1973) Role of Diffusion Boundary Layer in Contact Inhibition of Growth. *Nature, Lond.*, **246**, 200.

van den Brenk, H. A. S., Sharpington, C. & Orton, C. (1973*a*) Macrocolony Assays in the Rat of Allogeneic Y-P388 and W-256 Tumour Cells Injected Intravenously: Dependence of Colony Forming Efficiency on Age of Host and Immunity. *Br. J. Cancer*, **27**, 134.

van den Brenk, H. A. S., Burch, W. M., Orton, C. & Sharpington, C. (1973*b*) Stimulation of Clonogenic Growth of Tumour Cells and Metastases in the Lungs by Local X-radiation. *Br. J. Cancer*, **27**, 291.

van den Brenk, H. A. S., & Kelly, H. (1973*c*) Stimulation of Growth of Metastases by Local X-irradiation in Kidney and Liver. *Br. J. Cancer*, **28**, 349.

van den Brenk, H. A. S., Kelly, H. & Orton, C. (1974) Reduction by Anti-Inflammatory Corticosteroids of Clonogenic Growth of Allogeneic Tumour Cells in Normal and Irradiated Tissues of the Rat. *Br. J. Cancer*, **29**, 365.

van den Benk, H. A. S. & Upfill, J. (1958) Heterologous Growth of Ehrlich Ascites Tumour in Histamine-depleted Rats. *Aust. J. Sci.*, **21**, 20.

Willis, R. A. (1948) *Pathology of Tumours*. London: Butterworth & Co.

Willis, R. A. (1950) *Principles of Pathology*. London: Butterworth & Co.

WITHERS, H. R. & MILAS, L. (1973) Influence of Pre-irradiation of Lung on Development of Artificial Pulmonary Metastases of Fibrosarcoma in Mice. *Cancer Res.*, **33**, 1931.

WOOD, S. J., HOLYOKE, E. D. & YARDLEY, J. H. (1961) Mechanisms of Metastasis Production by Blood-borne Cancer Cells. *Can. Canc. Conf.*, 1961, Vol 4. London: Academic Press.